# The Mental Wellbeing of Child and Adolescent Mental Health Service (CAMHS) Workers in England: A Cross-Sectional Descriptive Study Reporting Levels of Burnout, Wellbeing and Job Satisfaction

**DOI:** 10.3390/healthcare12040430

**Published:** 2024-02-07

**Authors:** Silvana Mareva, Beth Chapman, Rebecca Hardwick, Charlotte Hewlett, Siobhan Mitchell, Amy Sanders, Rachel Hayes

**Affiliations:** 1 Faculty of Health and Life Sciences, University of Exeter, Exeter EX1 2LU, UK; s.m.mareva@exeter.ac.uk (S.M.); beth.chapman@nhs.net (B.C.); c.hewlett@exeter.ac.uk (C.H.); s.b.mitchell@exeter.ac.uk (S.M.); a.sanders@exeter.ac.uk (A.S.); 2 Peninsula Medical School, Faculty of Health, University of Plymouth, Plymouth PL6 8BH, UK; rebecca.hardwick@plymouth.ac.uk

**Keywords:** mental health, job satisfaction, burnout, wellbeing, healthcare

## Abstract

In the UK, there has been a notable increase in referrals to specialist children’s mental health services. This, coupled with shortages of qualified staff, has raised concerns about the escalating occupational stress experienced by staff in this sector. In this brief report, we present cross-sectional quantitative data from 97 staff members working in one Child and Adolescent Mental Health Service (CAMHS) in the UK during spring 2023, reporting on their wellbeing, job satisfaction, and burnout. Our findings reveal that over a third of CAMHS staff experienced moderate or high levels of work-related burnout; 39% reported moderate or high levels of personal burnout, but levels of client-related burnout were much lower (13%). Both work- and client-related burnout showed a robust negative relationship with job satisfaction, with higher burnout predicting lower levels of job satisfaction. Only a small proportion of respondents reported high levels of wellbeing, with about a quarter experiencing levels of wellbeing that can be considered indicative of mild or clinical depressive symptoms. Whilst these results are from a small sample in one area of the UK, they present an important snapshot of CAMHS staff wellbeing and are discussed in the context of similar trends reported in the wider NHS sector.

## 1. Introduction

In recent years, Child and Adolescent Mental Health Services (CAMHS) have grappled with a surge in referrals and an increase in the complexity of cases [1]. Nationally representative surveys estimate that about 1 in 5 children and young people aged 8 to 25 years have a probable mental health disorder [1]. These growing demands have been met with insufficient resources, with numerous reports concluding that staff shortages and inadequate funding mean that CAMHS services provide heavily restricted access to treatment to only a small subgroup of children and young people [2,3]. This has also led to raising levels of occupational stress among CAMHS workers, which in turn plays a pivotal role in influencing service quality, safety and staff wellbeing [4,5]. While occupational stress is often experienced on an individual level, top–down system changes are needed to transform the landscape of mental health services and promote the mental wellbeing of CAMHS workers [6]. 

Working in the mental health sector often carries high emotional demand, with research showing staff in this sector report poorer wellbeing than staff in other healthcare sectors [7]. Recent work from Ireland has shown high levels of burnout in both clinical and non-clinical CAMHS staff, with 57.6% reporting moderate or high levels of work-related burnout and 52.9% reporting moderate or high levels of personal burnout [4]. Looking specifically at consultant child psychiatrists working at CAMHS pre-pandemic, 75% reported moderate or high levels of work-related burnout and 72.3% moderate or high levels of personal burnout [8]. Notably, levels of client-related burnout were substantially lower in this group (26.9%). This discrepancy was not observed in the mixed sample of both clinical and non-clinical CAMHS staff, where about half of respondents reported high levels of client-related burnout and no significant differences were found between the two groups [4]. However, both studies relied on relatively small samples (52 consultants and 59 clinical and non-clinical) and further research is needed to explore potential differences between the experiences of clinical and non-clinical staff.

The high levels of occupational stress among CAMHS workers extend beyond the Irish context and are corroborated by another pre-pandemic study of Norwegian CAMHS therapists. It reported that 70% of respondents had medium-to-high levels of burnout and one-third reported a high level of intention to leave their job in the current or near future [9]. These patterns were not explained by individual factors such as age, job tenure or educational background. Qualitative work exploring the reasons cited for the high levels of burnout among child and adolescent consultant psychiatrists identified the lack of skilled staff and funding, along with widespread public misunderstanding of CAMHS’ remit as essential factors contributing to occupational wellbeing [6]. 

Stress and poor mental health were common concerns among CAMHS workers pre-pandemic. Similarly, a survey looking at the wellbeing of CAMHS staff amidst the pandemic found significantly lower levels of good mental health in comparison to population norms, with 17% of clinicians meeting criteria for a heightened risk of depression [10]. Additionally, a recent review by Wintour [11] on staff experiences in CAMHS highlighted elevated stress levels, insufficient resources, heavy workloads, and low job satisfaction. While all studies in the review indicated high emotional exhaustion, not all met the criteria for staff burnout.

Given the growing evidence about concerning levels of occupational stress in this group, it is paramount that more research considers contributing factors and seeks ways to enhance the workplace and support staff. In a study by Parry et al. [12], the focus was on a new integrated community mental health service and the experiences of staff working in this service. Essential elements contributing to staff wellbeing in the workplace were identified, including peer support, emotionally supportive leadership, supervision fostering career development and personal growth, and the availability of safe workspaces. Further research is needed to identify and promote infrastructural changes that can support the mental wellbeing of staff working in the mental health sector.

### Objectives of the Current Study

The cross-sectional quantitative data presented in this study were gathered with the primary aim of investigating staff attitudes towards integrating a novel nature-based approach into one UK CAMH service. Considering the increasing evidence supporting the positive relationship between contact with nature and wellbeing [13], the adoption of nature-based approaches could potentially benefit both staff and client wellbeing [14]. Early scoping conversations with staff in this CAMH service suggested a high prevalence of burnout and job dissatisfaction. However, there is a lack of post-pandemic data on the wellbeing and occupational stress experienced by the UK CAMHS workforce. To address this gap, we collected data on levels of burnout, wellbeing, and job satisfaction and explored their interrelationships and potential variations based on demographic and work-related factors (i.e., sex, age, clinical vs. non-clinical professional role, and working pattern). The objectives of this research were as follows:

(1) Assess the levels of staff burnout, wellbeing, and job satisfaction experienced in one UK CAMH service and how these are interrelated.
(2) Explore whether they differ by demographic and work-related factors (i.e., sex, age, clinical vs. non-clinical professional role, and working pattern).

Information regarding the use of nature-based approaches is not included in the current manuscript and will be analysed and reported separately.

## 2. Materials and Methods

### 2.1. Recruitment

The study was conducted in a community CAMH service in the UK that provides mental healthcare for children and families experiencing a range of difficulties. The sample was an opportunistic sample since all clinical and non-clinical staff working in the service were invited to participate in a short online survey administered through Qualtrics. The survey covered respondent demographics, burnout, wellbeing, and job satisfaction. We also asked questions about staff experience and attitudes towards nature-based approaches in child and adolescent mental health, and levels of nature connectedness, which are not included in this short report. The data were collected between February and March 2023. Respondents were offered a GBP 5 bank transfer or a shopping voucher as compensation for their time. Approval for this study was obtained from the Health Research Authority (IRAS project ID: 323703). Written consent to participate in the survey was obtained from all survey respondents. 

### 2.2. Measures

#### 2.2.1. The Copenhagen Burnout Inventory (CBI)

The CBI is an open-access validated questionnaire [15] assessing three domains of burnout: personal (six questions), work (seven questions), and client (six questions). The CBI has been used with many different health care professions to measure burnout across several different countries and has substantial validity evidence [16,17,18,19]; the Cronbach alphas for the subscales have been calculated as 0.85–0.87 [15], indicating good internal consistency. In the current study, the Cronbach alpha reliability coefficients of the CBI subscales were also high (personal α = 0.89; work-related α = 0.87; and client-related α = 0.90). The personal burnout scale is designed to provide a general index of the degree of physical and psychological fatigue and exhaustion experienced by the person as a whole, rather than a specific reflection of burnout related to their personal life only. The work burnout scale captures the exhaustion and fatigue directly associated with a participant’s job and work responsibilities, whilst the client scale focuses on specific challenges and stressors perceived by the respondents to arise from working directly with clients and interacting with service users. Twelve items have a frequency-based response format along a five-point Likert scale ranging from 100 (always), 75 (often), 50 (sometimes), 25 (seldom) and 0 (never/almost never). Seven items use response categories according to intensity, ranging from “a very low degree” to “to a very high degree”. Typical items from each scale are: “how often do you feel tired” (personal), “do you feel burnt out because of your work” (work-related) and “do you find it hard to work with clients” (client-related). According to the developers of the CBI and their definition of burnout, scores of 50 to 74 are considered moderate, 75–99 is considered high, and a score of 100 is considered severe burnout [20], and this definition has been used in many other studies [4,8,16,17,18].

#### 2.2.2. Short Warwick–Edinburgh Mental Wellbeing Scale (SWEMWBS)

The SWEMWBS is a validated questionnaire consisting of 7 items that assess respondent wellbeing on a 5-point Likert scale [21]. Construct and external validity have been established for the SWEMWBS in diverse populations [21,22,23,24,25] using multiple methods, and test–retest reliability has been confirmed [26]. In this study, the Cronbach alpha reliability coefficient of the SWEMWBS was high (α = 0.83). The SWEMWBS is free to use, but you need to ask for permission before you begin using the SWEMWBS by completing a registration form on the SWEMWBS website [27]. Higher scores indicate greater levels of wellbeing. The SWEMWBS has a mean of 23.5 and a standard deviation of 3.9 in UK general population samples [21]. This means that 15% of the population can be expected to have a score of >27.4; therefore, the established cut point to indicate high wellbeing was set at 27.5. Similarly, 15% of the population can be expected to have a score <19.6; therefore, the established cut point to indicate low wellbeing was set at 19.5. The SWEMWBS is scored by first summing the scores for each of the seven items, which are scored from 1 to 5. The total raw scores are then transformed into metric scores using the SWEMWBS conversion table [22]. Scores below 20 are considered to indicate low levels of wellbeing, scores in the range between 20 and 27 indicate moderate levels of wellbeing, and scores of 28 and above indicate high levels of wellbeing [27]. The SWEMWS score can also be benchmarked against the Patient Health Questionnaire (PHQ-9) [28] to assess the level of depressive symptoms currently experienced. SWEMWBS scores between 18 and 20 correspond to possible mild depression or anxiety; scores of 18 or less correspond to probable clinical depression or anxiety; and scores of >20 correspond to scores indicating no depressive symptoms [29].

#### 2.2.3. Short Index of Job Satisfaction (SIJS)

The SIJS is an open access short, validated questionnaire [30] comprising five questions rated on a five-point Likert scale (e.g., “I feel fairly satisfied with my present job”). The SIJS has demonstrated good internal validity in previous studies [31,32]. Total scores can range between 5 and 25 with no cut-off scores. Higher scores indicate higher levels of job satisfaction. Whilst this measure does not have established UK population norms, it has good psychometric properties and, with 5 items, does not impact participant burden. In this study, the Cronbach alpha reliability coefficient of the SIJS was high (α = 0.86) 

### 2.3. Data Analysis

The data analysis plan focused on exploring the two main research objectives: (1) assess the levels of staff burnout, wellbeing, and job satisfaction experienced in one UK CAMH service and how these are interrelated; (2) explore whether they differ by demographic and work-related factors (i.e., sex, age, clinical vs. non-clinical professional role, and working pattern). To address the first research objective, we reported average levels of burnout, wellbeing, and job satisfaction and interpreted them against established benchmarks (where these were available). To explore the relationship between these three variables, Pearson correlations were estimated. Potential links with sex (dichotomous: male vs. female), age (categorial: under 25, between 25 and 35, between 35 and 44, between 45 and 54, between 55 and 64, and 65 and over), clinical vs. non clinical professional role (dichotomous: clinical staff vs. non-clinical staff), and working pattern (dichotomous: part-time vs. full-time) were investigated using a series of t-tests for dichotomous variables and ANOVA for the categorical variable. All analyses were conducted in R version 4.2.0 using psych package version 2.3.3.

## 3. Results

### 3.1. Participants

A total of 97 CAMHS employees completed the survey (83 female, 13 male, 1 gender-fluid; note that in order to maintain participant anonymity, only comparisons between males and females are reported due to a single data point in the gender-fluid group), of whom 59 were employed full-time and 29 worked part-time (9 respondents did not provide information about their working patterns). Participants represented a mix of clinical and non-clinical professions (professions reported using a free text response format: other clinical: N = 36; clinical associate psychologist: N = 16; nurse: N = 12; non-clinical: N = 12; doctor: N = 8; clinical psychologist: N = 5; psychology intern: N = 3; other (occupational therapy, health and social care): N = 5). The age composition of the sample is shown in Table 1. The breakdown of time staff had worked at the trust was the following: less than 1 year, N = 36; between 1 and 2 years, N = 6; between 2 and 5 years, N = 34; between 5 and 10 years, N = 12; between 10 and 15 years, N = 5; over 15 years, N = 4. 

### 3.2. Copenhagen Burnout Inventory (CBI)

**Figure 1:**
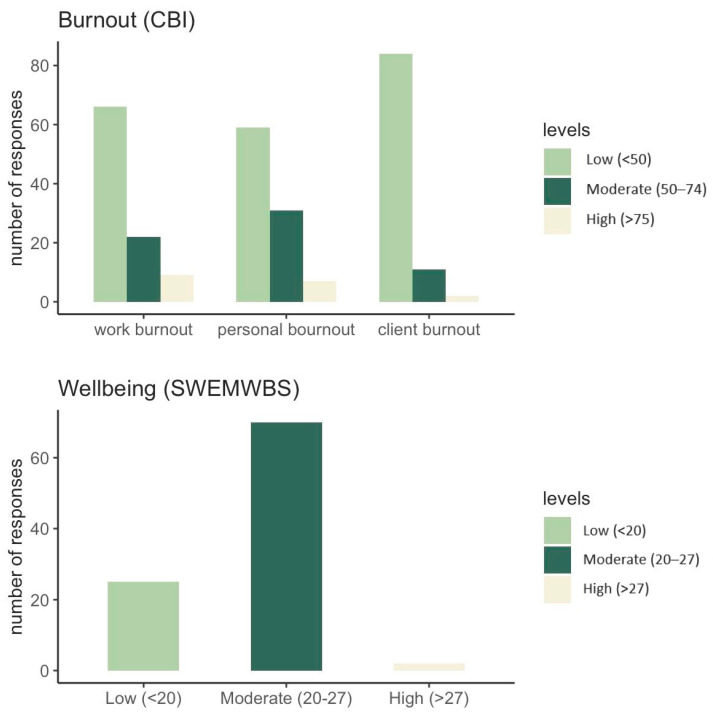
Number of participant responses on the Copenhagen Burnout Inventory (CBI) and Short Warwick–Edinburgh Mental Wellbeing Scale (SWEMWBS) classified as low, moderate, and high levels of burnout/wellbeing.

The average CBI domain scores are shown in Table 1. Figure 1 indicates the proportion of respondents reporting low, moderate, or high burnout. About a third (32%) of respondents reported moderate (23%) or high (9%) work-related burnout; 39% reported moderate (32%) or high (7%) personal-related burnout. For client-related burnout, the proportion of responses classed as moderate (11%) or high (2%) was substantially lower (13%). No differences were observed between males and females across any of the CBI domains: Work: t = −0.65, *p*= 0.52; Personal: t = 1.32, *p* = 0.19; Client: t = −0.05, *p* = 0.96 (see Table 1). There were no significant differences between clinical and non-clinical staff (Work: t = 1.02, *p* = 0.29; Personal: t = 0.20, *p* = 0.84; Client: t = −0.20, *p* = 0.85; see Table 1), between those working full-time and part-time (Work: t = 0.67, *p* = 0.50; Personal: t = 1.25, *p* = 0.21; Client: t = −1.07, *p* = 0.29; see Table 1), or across age groups (Work: F = 0.75, *p* = 0.59, Personal: F = 0.49, *p* = 0.78; Client: F = 1.41, *p* = 0.23; see Table 1). 

### 3.3. SWEMWBS

The average SWEMWBS score was 22.47 (SD = 2.81), which is within the moderate range relative to UK averages. Figure 1 shows the breakdown according to the cut-offs proposed in the previous literature [27]. Most (72%) respondents reported moderate levels of wellbeing, 26% reported low levels of wellbeing, and only 2% reported high levels of wellbeing. Considering benchmarks against PHQ-9, in this sample, we found that 5% of respondents (5 out of 97) reported wellbeing levels indicative of probable clinical depression and 21% (20 out of 97) scored in the range of possible mild depression [29]. This suggests that about a quarter of the sample were experiencing mild-to-severe depressive symptoms. No significant differences were observed across male and female participants (t = 0.03, *p* = 0.98, Table 1), those working part-time or full-time (t = −1.42, *p* = 0.16, Table 1), clinical and non-clinical staff (t = −0.40, *p* = 0.70, Table 1), and across age groups (F = 0.88, *p* = 0.50). 

### 3.4. SIJS

The mean in this sample was 19.93 (SD = 3.31). No differences were observed across males and females (t = −0.49, *p* = 0.63, Table 1), those working part-time and full-time (t = 0.61, *p* = 0.55, Table 1), clinical and non-clinical staff (t = −0.53, *p* = 0.61, Table 1), or across age groups (F = 0.14, *p* = 0.98). The proportion of participants endorsing each response option across the five SIJS questions is presented in Supplementary Table S1. Overall, about 70% of respondents endorsed that they either agreed or strongly agreed with statements such as “I feel fairly satisfied with my present job” and “I find real enjoyment in my work”.

### 3.5. Relationship between Burnout, Wellbeing, and Job Satisfaction

**Figure 2:**
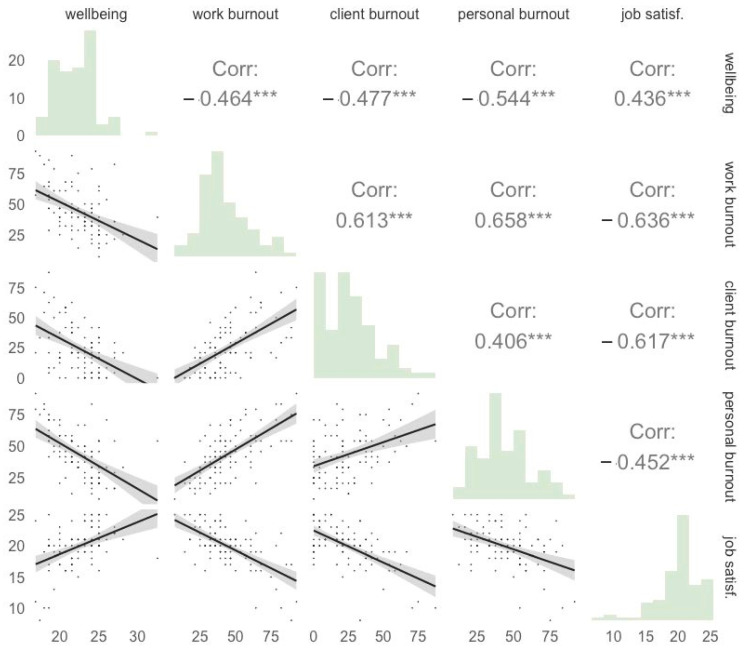
Histograms and Pearson correlations across levels of wellbeing, job satisfaction and burnout. Note. The diagonal shows a histogram of the distribution of each measure. The bottom panel displays scatterplots across pairs of measures and the upper panel displays the Pearson correlation coefficients. Wellbeing = SWEMWBS total metric score; work, client, and personal burnout = total score of the respective CBI scale; job satisf. = SIJS total score *** = *p* < 0.001.

The Pearson correlations across levels of job satisfaction, burnout, and wellbeing are shown in Figure 2. Job satisfaction was negatively related to work, personal, and client burnout (all *p* < 0.005) and positively related to wellbeing. Wellbeing levels were negatively related to all three domains of burnout (all *p* < 0.005).

## 4. Discussion

This study provides insights into the wellbeing, burnout, and job satisfaction levels among the CAMHS workforce in a UK Trust. The results indicate that about a third of this workforce is experiencing moderate-to-high levels of work-related burnout, and a quarter reported levels of wellbeing indicative of potential depressive symptoms. Burnout and wellbeing levels did not systematically vary across demographic and work environment factors, including whether staff worked clinically or not. Expectedly, both work- and client-related burnout showed a strong negative relationship with job satisfaction, indicating that higher burnout scores were predictive of lower levels of job satisfaction. The proportion of staff reporting high client-related burnout was considerably lower than those reporting work-related or personal burnout. The relatively low client-related burnout scores reported in comparison to levels of personal and work-related burnout are noteworthy and consistent with the experiences reported by other healthcare professionals in the UK and worldwide [33,34]. 

The work-related burnout levels observed in the current study are comparable with national statistics for the NHS workforce, where similarly, just over a third report feeling burnt out because of their work [35]. Our results suggest that this UK sample reported lower levels of burnout than those reported in similar CAMHS samples in Ireland and Norway [4,8,9], a discrepancy which could be due to sampling or context-specific factors. Consistent with previous findings that the work environment and processes seem to have more influence on the burnout of NHS staff than individual characteristics, we did not observe any systematic variation based on any of the demographic factors explored [36]. System-level factors including human resources, adequacy of services, professional relationships, socio-political factors, and public perception merit further investigation as potential key factors that can contribute to the occupational stress of staff that support children and young people’s mental wellbeing [6]. Further research should systematically explore how these factors may be impacting working conditions across this sector with the goal to alter those working environments that promote job dissatisfaction and burnout. 

Whilst the UK sample is reporting lower levels of burnout relative to estimates from Ireland and Norway, the fact that a third of the sample is experiencing moderate-to-high work-related and personal burnout should still be seriously considered by service managers, considering well-established links between levels of burnout and the amount of time healthcare professionals are not able to work due to sickness [37]. These levels of burnout are also important when we consider their relationship to job satisfaction and wellbeing. Our data show a clear relationship between burnout, wellbeing, and job satisfaction, but further research is warranted to explore the direction of these associations. In terms of wellbeing, we also note that about the quarter of respondents experienced levels of wellbeing indicative of mild or severe depressive symptoms. This percentage is somewhat higher than post-pandemic estimates of prevalence in the general UK population, with most recent statistics suggesting that 16% of adults experience moderate-to-severe depressive symptoms [38]. Whilst our findings suggest elevated levels of poor mental health among CAMHS professionals, we recommend that further research explores this with larger samples and direct assessments of depressive symptoms.

### Strengths and Limitations

A key strength of the study is its use of well-validated measures, which facilitate national and international comparisons. However, the sample was small and self-selecting, so there may be under- or over-representation of CAMHS workers experiencing burnout and low job satisfaction, which may limit the generalisability of the current findings. Currently, UK national norms for the SIJS are not available, making it difficult to benchmark job satisfaction levels. Given that the primary focus of the survey was to explore nature-connectedness and the use of nature-based interventions, it is likely that our respondents were more likely to be those who have an interest in this area. We did not observe any significant demographic differences across levels of burnout, wellbeing, and burnout, which may still become apparent in larger samples.

## 5. Conclusions

The results suggest that about a third of the CAMHS staff surveyed are experiencing moderate-to-high levels of work-related burnout, levels comparable to national averages across the NHS workforce. We also observed that about a quarter of CAMHS workers reported levels of wellbeing indicative of mild-to-severe depression, a prevalence which is elevated relative to the UK population average. Burnout and wellbeing levels did not systematically vary across demographic and work environment factors, including whether staff worked in a clinical role or not. We observed robust associations across levels of burnout, wellbeing, and job satisfaction, with higher levels of burnout predicting lower wellbeing and higher job dissatisfaction. However, it should be noted that the sample size was small and self-selecting, which may limit the generalisability of these findings. There is considerable scope for more research into how levels of occupational stress might be addressed to promote the mental wellbeing of CAMHS workers, which has important implications for service quality and patient outcomes.

## Figures and Tables

**Table 1:** Levels of work, personal, and client burnout, wellbeing, and job satisfaction across different demographic and work circumstances groups.

Group		Work Burnout (CBI)			Personal Burnout (CBI)			Client Burnout (CBI)			Wellbeing (SWEMWBS)			Job Satisfaction (SIJS)		
	N	MIN–MAX	M	SD	MIN–MAX	M	SD	MIN–MAX	M	SD	MIN–MAX	M	SD	MIN–MAX	M	SD
Full sample	97	7.1–92.9	44.1	18.5	8.3–91.7	43.6	18.6	0.0–87.5	24.5	20.6	16.9–32.6	22.5	2.8	8.0–25.0	19.9	3.3
**Work pattern**																
part-time	29	14.3–89.3	43.6	19.3	16.7–70.8	40.8	15.7	0.0–62.5	28.9	20.3	19.3–27.0	22.9	2.4	13.0–25.0	19.5	2.9
full-time	59	17.9–92.9	46.4	18.2	12.5–92.3	46.1	19.7	0.0–87.5	23.9	20.4	16.9–32.6	22.0	3.0	8.0–25.0	19.9	3.6
**Professional group**																
clinical staff	85	14.3–89.3	45.0	17.9	12.5–83.3	43.7	18.2	0.0–87.5	24.3	19.6	16.9–32.6	22.4	2.9	8.0–25.0	19.8	3.2
non-clinical staff	12	7.1–92.9	37.8	21.6	8.33–91.7	42.4	22.25	0.0–75.0	25.7	23.3	16.9–25.0	22.7	2.3	11.0–25.0	20.5	4.1
**Age**																
under 25	8	25.0–78.6	41.5	16.4	20.8–83.3	47.9	22.3	4.2–54.2	22.9	16.2	20.0–25.0	22.9	1.9	17.0–22.0	19.8	1.5
between 25 and 34	27	17.9–89.3	47.6	18.9	12.5–79.2	42.1	116.0	0.0–87.5	28.2	19.6	16.9–27.0	22.0	2.7	10.0–25.0	20.2	3.6
between 35 and 44	25	17.9–89.3	41.6	14.7	16.7–75.0	44.2	16.6	0.0–66.7	18.7	16.9	17.4–28.1	22.6	2.6	8.0–25.0	20.1	3.2
between 45 and 54	28	7.1–92.9	44.9	21.7	8.3–91.7	43.3	21.0	0.0–75.0	26.2	22.8	16.9–32.6	22.9	3.3	10.0–25.0	19.5	4.0
between 55 and 64	7	17.9–67.9	44.9	19.2	20.8–79.2	47.6	24.5	8.3–62.5	32.1	21.8	18.0–25.0	21.1	2.5	17.0–22.0	19.9	1.9
65 and over	2	14.3–35.7	25.0	15.2	16.7–37.5	27.1	14.7	0.0–4.2	2.08	2.95	22.4–27.0	24.7	3.3	19.0–22.0	20.5	2.1
**Sex**																
male	13	17.9–89.3	47.3	21.5	16.7–75.0	37.5	16.8	4.17–58.3	25.0	15.8	17.4–27.0	22.4	2.9	8.0–24.0	19.5	3.9
female	83	7.1–92.9	43.6	18.2	8.3–91.7	44.8	18.8	0.0–87.5	24.7	20.6	16.9–32.6	22.4	2.9	10.0–25.0	20.0	3.2

Note. For all burnout scales, a total score below 50 indicates low levels of burnout; a total score of 50–74 indicates moderate burnout; and a total scale score of 75–99 indicates high level of burnout [16]. A SWEMWBS total metric score below 20 is considered low wellbeing, SWEMWBS total metric score between 20 and 27 is considered moderate wellbeing, and score above 27 is considered high level of wellbeing [27].

## Data Availability

The data that support the findings of this study are available from the corresponding author, (R.H.), upon reasonable request.

## Supplementary Materials

The following supporting information can be downloaded at: https://www.mdpi.com/article/10.3390/healthcare12040430/s1; Table S1: Percentage of respondents endorsing each one of the response categories across the five questions of the Short Index of Job Satisfaction (SIJS).
