# Supplementary material for: The Mental Wellbeing of Child and Adolescent Mental Health Service (CAMHS) Workers in England: A Cross-Sectional Descriptive Study Reporting Levels of Burnout, Wellbeing and Job Satisfaction
Source: Healthcare (Basel). 2024 Feb 7;12(4):430. doi: 10.3390/healthcare12040430 (PMC10888124; doi:10.3390/healthcare12040430)
Supplement: Supplementary file 1 [file healthcare-12-00430-s001.zip › healthcare-2812052-supplementary.pdf]

Supplementary Table S1. Percentage of respondents endorsing each one of the response categories across the five questions of the Short Index of Job Satisfaction (SIJS).

Question	Strongly Disagree	Disagree	Undecided	Agree	Strongly Agree
I feel fairly satisfied with my present job	1%	7%	20%	53%	20%
Most days I am enthusiastic about my work	1%	5%	14%	59%	21%
Each day at work seems like it will never end	24%	56%	12%	7%	1%
I find real enjoyment in my work	0%	8%	8%	58%	26%
I consider my job to be rather unpleasant	38%	53%	3%	6%	0%
